# The mechanism of electroacupuncture-mediated improvement in Parkinson’s disease by inhibiting ferroptosis through activating the Nrf2/GPX4 signal pathway

**DOI:** 10.3389/fnagi.2025.1551404

**Published:** 2025-05-07

**Authors:** Min Wang, He-Sheng Zheng, Wei-Liang Ye, Jin-Dong Mao, Kun Zhang, Le Yang, Ming-Gao Zhao, Shui-Bing Liu, Rui Liu, Yu-Mei Wu

**Affiliations:** ^1^Department of Pharmacology, School of Pharmacy, Fourth Military Medical University, Xi’an, China; ^2^Department of Acupuncture-Moxibustion-Massage, Shaanxi University of Chinese Medicine, Xi’an, China; ^3^Department of Pharmaceutics, School of Pharmacy, Fourth Military Medical University, Xi’an, China; ^4^Department of Pharmacy, Tangdu Hospital, Fourth Military Medical University, Xi’an, China; ^5^Department of Rehabilitation Medicine, Tangdu Hospital, Fourth Military Medical University, Xi’an, China

**Keywords:** Parkinson’s disease, ferroptosis, electroacupuncture, nuclear factor erythroid 2-related factor 2, glutathione peroxidase 4, substantia nigra

## Abstract

**Introduction:**

Ferroptosis, an iron-dependent regulated cell death pathway, shares several features of Parkinson’s disease (PD) physiopathology, and efficient neuroprotective therapies are required to prevent DAergic neuron death initiated by ferroptosis. Electroacupuncture (EA), a treasure of Traditional Chinese Medicine, exerted therapeutic effects against PD to avoid the side effects of dopamine (DA)-based therapies. However, its underlying mechanisms still need to be fully understood.

**Methods:**

MPTP-induced PD mice were treated with EA to evaluate its neuroprotective effects. Behavioral assessments, histopathological analysis of DAergic neurons, and quantification of ferroptosis biomarkers-including malondialdehyde (MDA), 4-hydroxynonenal (4-HNE), iron, glutathione (GSH), and mitochondrial integrity-were performed. Protein expression levels of SLC7A11, GPX4, ferritin heavy chain 1 (FTH1), and nuclear factor erythroid 2-related factor 2 (Nrf2) were analyzed via immunoblotting. To validate pathway specificity, the Nrf2 inhibitor trigonelline (AT) was co-administered with EA.

**Results:**

EA treatment significantly mitigated MPTP-induced DAergic neuron loss and motor deficits. Mechanistically, EA suppressed ferroptosis by reducing lipid peroxidation and iron accumulation while restoring GSH levels. It upregulated ferroptosis-suppressive proteins SLC7A11, GPX4, FTH1, and Nrf2, alongside ameliorating mitochondrial dysfunction. Crucially, AT administration abolished EA’s protective effects, confirming Nrf2 pathway dependency.

**Discussion:**

These findings demonstrate that EA exerts neuroprotection in PD by inhibiting ferroptosis through activation of the Nrf2/SLC7A11/FTH1/GPX4 signaling axis. This study not only elucidates a novel mechanism underlying EA’s efficacy in PD but also highlights ferroptosis modulation as a therapeutic strategy, bridging traditional medicine with molecular pathophysiology. This study has provided new ideas for exploring the mechanism of EA in PD treatment.

## 1 Introduction

Parkinson’s disease (PD), the second most common neurodegenerative disorder after Alzheimer’s disease, is characterized by the progressive degeneration of dopaminergic neurons in the substantia nigra (SN) (Tian et al., 2020). Different mechanisms of cell death contribute to PD pathogenesis, including apoptosis, necrosis, pyroptosis, and ferroptosis. Among these mechanisms, ferroptosis is a newly identified non-apoptotic form of regulated cell death and is characterized by Fe^2+^-dependent lethal accumulation of lipid peroxides, and the pathophysiology of ferroptosis has several features that are consistent with that of PD (Stockwell et al., 2017), which suggests the potential association between PD and ferroptosis (Guiney et al., 2017; Hambright et al., 2017; Mahoney-Sanchez et al., 2021). Furthermore, ferroptosis is initiated by abnormal iron metabolism, imbalance between the production and degradation of lipid reactive oxygen species (ROS), loss of glutathione (GSH), and decrease in mitochondrial membrane potential (MMP) (Dixon et al., 2012; Ou et al., 2022).

Ferroptosis is driven by the loss of activity of the lipid repair enzyme glutathione peroxidase 4 (GPX4), a GSH-dependent enzyme (Seibt et al., 2019). GSH acts as a cofactor for GPX4 and maintains the level of GPX4 through the exchange of glutamate and cystine via the cystine/glutamate antiporter system Xc-; thus, Xc- is involved in GSH biosynthesis. GPX4 uses two GSH molecules to safely reduce phospholipid hydroperoxides to their corresponding lipid alcohols, producing H_2_O and glutathione disulfide as byproducts. In addition, GPX4 reduces lipid hydroperoxides to non-toxic lipid alcohols (Crielaard et al., 2017), thus limiting the propagation of lipid peroxidation within the membrane and preventing the Fe^2+^-dependent production of ROS (Ward et al., 2014), particularly lipid hydroperoxides. Inhibition of GSH synthesis and system Xc-, or direct inactivation of GPX4, ultimately results in the accumulation of toxic lipid ROS and ferroptotic cell death (Dixon et al., 2012; Tian et al., 2020; Xie et al., 2016). Furthermore, ferritin heavy chain 1 (FTH1) can reverse ferroptosis by regulating iron storage, trapping Fe^2+^ ions inside the cell, and converting them to Fe^3+^, thus reducing ROS production (Do Van et al., 2016; Wang et al., 2022). Clinical trials on PD have shown that the anti-ferroptotic iron chelator offers neuroprotective support through slowing the disease progression and improving the motor function, which highlights the key role of iron in ferroptosis (Wu et al., 2017). Moreover, the importance of erythroid 2-related factor 2 (Nrf2), an antioxidant transcription factor, in ferroptosis is elucidated by the fact that almost all genes that have been implicated to date in ferroptosis are transcriptionally regulated by Nrf2 (Shah et al., 2023). These genes include genes involved in GSH regulation (synthesis and cysteine supply *via* system Xc-, glutathione reductase, and GPX4), NADPH regeneration, which plays a crucial role in GPX4 activity, and iron regulation (Ou et al., 2022; Xu et al., 2023). Nrf2 knockout results in impaired mitochondrial function, whereas Nrf2 activation enhances mitochondrial function and resistance to stressors (Greco and Fiskum, 2010; Holmstrom et al., 2016; Neymotin et al., 2011). Therefore, Nrf2 plays a crucial role in ferroptosis as it regulates numerous genes that are central to ferroptosis. Enhancing Nrf2 signaling has neuroprotective effects in PD and prevents ferroptosis.

As discussed earlier, PD can be improved by inhibiting ferroptosis as it reduces the loss of DAergic neurons associated with PD. Electroacupuncture (EA), an invaluable Traditional Chinese Medicine approach, has increasingly become an important choice for patients due to its safe, reliable, and inexpensive therapeutic effects, as well as low use of medication and thus significantly reduced side effects. EA treatment has been shown to resist PD symptoms in multiple ways. This study was carried out to investigate whether EA offers therapeutic effects on PD by inhibiting ferroptosis. The findings of this study indicate that ferroptosis is associated with the loss of DAergic neurons in the substantia nigra (SN) of PD model mice following 1-methyl-4-phenyl-1,2,3,6-tetrahydropyridine (MPTP) insult and that EA administration alleviates the motor dysfunction of PD mice by inhibiting ferroptosis. Further results have confirmed that EA increases Nrf2 expression, followed by the upregulation of the key molecules SLC7A11, FTH1, and GPX4 to improve mitochondrial dysfunction, thus relieving PD symptoms by inhibiting the ferroptosis of DAergic neurons. These findings shed light on EA-mediated improvement in PD by inhibiting the ferroptosis signaling Nrf2/SLC7A11/FTH1/GPX4 pathway and provide a clear understanding of the previously unrecognized anti-ferroptotic mechanism of EA in treating PD.

## 2 Materials and methods

### 2.1 Materials

MPTP hydrochloride (catalog no. S4732) was purchased from Selleck.cn (Houston, TX, United States). The following antibodies were used in Western blot experiments: anti-GPX4 (1:1,000, Abcam, ab125066), anti-Nrf2 (1:1,000, ABclonal, A11159), anti-tyrosine hydroxylase (TH) (1:1,000, Cell Signaling Technology, #58844), anti-SLC7A11 (1:1,000, Abcam, ab216876), anti-FTH1 (1:250, Abcam, ab183781), and anti-*β*-actin (1:10,000, Sigma-Aldrich, A5441) antibodies. All secondary antibodies conjugated with horseradish peroxidase (HRP) were purchased from Santa Cruz Biotechnology (Santa Cruz, CA, United States). The antibodies used in immunofluorescent experiments were as follows: anti-TH (1:100, Cell Signaling Technology, 58844) and Alexa fluor-488/594 conjugated anti-rabbit (1,500, Invitrogen, A-21206/A-21207) antibodies. Malondialdehyde (MDA) Assay Kit (M496) and GSH Assay Kit (G263) were obtained from Elabscience Biotechnology (Wuhan, China), and alkaloid trigonelline (AT), an Nrf2 inhibitor, was obtained from MedChemExpress (Monmouth Junction, NJ, United States). AT was dissolved in 0.9% sterile saline to achieve the desired concentrations. The BCA Kit, M-PER Protein Extraction Buffer, and enhanced chemiluminescent solution (ECL) were obtained from Pierce (Rockford, IL, United States). Polyvinylidene difluoride (PVDF) membrane was purchased from Merck-Millipore. All chemicals were obtained from Sigma-Aldrich unless otherwise stated.

### 2.2 Experimental procedures

Male C57BL/6 mice (age 7–8 weeks, weight 20–30 g) obtained from the Experimental Animal Center of the Fourth Military Medical University (certificate no. 201000082, grade II) were used in experiments. All mice were raised under standard laboratory conditions with a 12-h light/dark cycle at 22 ± 1°C temperature and 55–60% humidity, with water and food provided *ad libitum*.

To determine the therapeutic effect of EA on PD and the mechanisms involved, 24 mice were randomly divided into five groups: control (Ctrl), MPTP, MPTP + sham EA, MPTP + EA, and MPTP + levodopa (L-DA) groups (*n* = 8 per group). The PD model was established by an intraperitoneal (i.p.) injection of MPTP (30 mg/kg) once daily for 5 days. The mice in the MPTP + sham EA group were subjected to non-acupoint stimulation for 7 days before MPTP injury. The mice in the MPTP + EA and MPTP + L-DA groups were subjected to EA treatment and L-DA in water intragastrically (20 mg/kg i.g.), respectively, for 7 days before MPTP injury, followed by an additional 5 days of respective treatment. The Ctrl group received an equal volume of saline. The two acupoints Baihui and Fengfu are positioned in the Du Meridian. In particular, Baihui is located at the midpoint of the line connecting the two ear tips, and Fengfu is located at the depression between the occiput and the atlas. Sterile stainless steel needles (0.35 mm diameter, 13 mm long) were inserted into the acupoints and connected to the EA instrument. Then, the acupoints were stimulated at 1 mA intensity and 5 Hz frequency for 30 min per day, for 12 days consecutively. One day after the last treatment, behavioral assessments were carried out using an open field test (OFT), rotarod test, traction test, and pole test. On the 14th day, all animals were euthanized after deep anesthesia for further study. All experiments were conducted in accordance with the principles established for the care and use of laboratory animals by the National Institutes of Health and were approved by the Animal Care and Use Committee of the Fourth Military Medical University. Behavior tests were carried out 30 min after the last treatment.

### 2.3 Open field test

The OFT was carried out to assess the autonomic motor ability of mice from each group, as described in a previous work (Yang et al., 2016). Briefly, each mouse was placed at the center of an open field apparatus (50 × 50 × 60 cm) and allowed to explore freely. A light level of *<* 50 lx was chosen as it has little influence on mouse behavior. The total distance traveled, suspended time, and upright time of the mice were recorded during a test period of 5 min using a video camera above the arena and analyzed using a video-tracking system (DigBehav system, Yishu Co., Ltd.). The arena was carefully cleaned with 70% alcohol and rinsed with water after each test.

### 2.4 Traction test

Mice were placed in the behavior room 12 h in advance to adapt to the new environment. The latency (in seconds) to fall from the metal wire grasped by the front paws was recorded, and the assessment depended on the time period that mice could remain on a metal wire within 3 min. The motor performance of the front paws was evaluated three times a day with 30-min intervals, and the average retention time of the three trials was calculated. The test was performed by a reviewer blinded to the animal groups.

### 2.5 Rotarod test

Motor coordination was evaluated using a rotating rod apparatus (Panlab Harvard Apparatus, Barcelona, Spain), as described in a previous study (Gao et al., 2020). Each mouse was given a 1-min trial on the rod (3.2 cm diameter), followed by speed accelerating from 4 to 40 rpm within 300 s. The latency (in seconds) to fall from the rolling rod was recorded, and the assessment depended on the time period that mice could remain on the rotating rod. The motor performance was evaluated three times a day with 30-min intervals, and the average retention time of the three trials was calculated. This test was performed by a reviewer blinded to the animal groups. The results were expressed as retention time on the rotating bar over the three test trials (mean ± standard error of the mean (SEM) in each group).

### 2.6 Pole test

The pole test was used to evaluate the coordination and balance ability of mice and was carried out at 15:00 p.m. on day 14. A ball was attached to the top of a vertical Polyvinyl chloride (PV) tube (1.0 cm diameter, 55 cm long) that was tightly wrapped with a double layer of gauze to prevent slipping. The base of the pole was covered with bedding to protect mice from injury. After acclimatization, mice were pretrained with the pole three times to ensure that all animals would turn their head down once they were put on the ball 3 days prior to the formal testing. During the pole test, each mouse was placed head upward on the ball, and the time for completing the turn of the head (t-turn) and the total time from the top to the bottom of the rod (t-total) were recorded. If a mouse failed to flip or slip completely, the time was recorded as 120 s. Each mouse was tested three times with a 2-min interval between each test, and the results were expressed as mean ± SEM in each group.

### 2.7 Immunofluorescence staining

After behavior assessments, mice were anesthetized with sodium pentobarbital (40 mg/kg, *i.p.*) following ethical principles and then perfused intracardially with saline followed by 4% paraformaldehyde (PFA) in 0.1 M phosphate buffer solution (PBS, pH 7.4). Sequential 30-μm-thick coronal sections of the SN (2.8–3.74 mm anterior to the Bregma) were collected in PBS as floating sections. The cryosections containing the SN were incubated in 5% bovine serum albumin (BSA) and 10% goat serum for 1 h to block non-specific binding after the wash and penetration at room temperature (RT). The slices were incubated with primary anti-TH antibody overnight at 4°C, followed by Alexa Fluor secondary antibody (Invitrogen, United States) incubation for 1 h at RT. Nuclei were counterstained with DAPI (4,6-diamidino-2-phenylindole). Fluorescent signals were photographed using confocal microscopy (Olympus, Japan). For TH^+^ cell analysis, Image J software (US National Institutes of Health, Bethesda, MD, United States) was used to calculate all the DAergic neurons in the SN region on serial sections at 100× magnification.

### 2.8 JC-1 staining

MMP was assessed using JC-1 staining, which was carried out using dissociated cells. Briefly, SN tissues were dissected and digested for 45 min at 36°C in a Petri dish with 2.5 mL papain solution (1 × EBSS, 0.46% d-glucose, 26 mM NaHCO_3_, 50 mM EDTA, 75 U/mL DNase I, 300 units of papain, and 2 mM L-cysteine) while bubbling with 5% CO_2_ and 95% O_2_. The tissue was washed four times with ovomucoid solution (1 × EBSS, 0.46% d-glucose, 26 mM NaHCO_3_, 1 mg/mL ovomucoid, 1 mg/mL BSA, and 60 U/mL DNase I) after the digestion process and mechanically dissociated with two fire-polished borosilicate glass pipettes. The bottom layer of concentrated ovomucoid solution (1 × EBSS, 0.46% d-glucose, 26 mM NaHCO_3_, 5.0 mg/mL ovomucoid, 5.5 mg/mL BSA, and 25 U/mL DNase I) was added to the cell suspension. The tubes were centrifuged at 300×*g* for 10 min at RT, and the resultant pellet was resuspended in D-PBS with 0.02% BSA and 13 U /mL of DNase I and filtered through a 20-μm mesh. The cells were collected in the working solution of JC-1 at 37°C for 20 min and were washed three times with cold-washing buffer. The stained cells were finally analyzed using a Coulter XL (Beckman Coulter, Brea, CA, United States) flow cytometer (Qi et al., 2021).

### 2.9 Western blot assay

The whole SN tissues of mice from each group were harvested, lysed using RIPA lysis buffer containing 1% protease inhibitor cocktail for 30 min, and centrifuged for 20 min at 4°C. Protein concentration in the samples was quantified using a BCA Assay Kit, followed by electrophoretic separation through SDS-PAGE. The protein samples were transferred to PVDF membranes and blocked with 5% BSA dissolved in Tris-buffered saline with 0.1% Tween 20 for 2 h at RT. The membranes were incubated overnight in the presence of primary antibodies against TH, Nrf2, FTH1, SLC7A11, and GPX4 at 4°C, with *β*-actin serving as the loading control. They were subsequently incubated with HRP-conjugated secondary antibodies for 1 h at RT. Finally, immunoblots were detected using ECL solution and analyzed using Image J software.

### 2.10 Determination of neurotransmitter levels using high-performance liquid chromatography (HPLC)

The SN tissue samples were weighted and homogenized in 0.1 M HClO_4_, incubated on ice for 1 h, and then centrifuged at 12,000×*g* for 20 min at 4°C. Homogenates were normalized using a Bradford assay, and the levels of dopamine (DA), 3,4-dihydroxyphenylacetic acid (DOPAC), and homovanillic acid (HVA) in each sample were quantified using HPLC. A Shim Pack C18 column (6.0 mm × 150 mm, 5 μm i.d.) was used for the separation. The mobile phase consisted of 0.1 M KH_2_PO_4_–methanol buffer (9:1 volume ratio) at the flow rates of 0.5 mL/min (10 min), 1 mL/min (2.5 min), 1.5 mL/min (18 min), and 0.5 mL/min (2 min) at RT. Under analytical conditions, 20 μL of the sample was taken. The excitation wavelength of the fluorescence detector was 254 nm, and the emission wavelength was 338 nm. The concentrations of DA, DOPAC, and HVA were expressed as ng/mg of tissue (Lee et al., 2019; Liu et al., 2018).

### 2.11 Quantification of iron levels

The Iron Assay Kit (Abcam) was used to quantify iron levels. SN tissues were harvested, washed with cold PBS, and then homogenized in 4 to 10 volumes of iron assay buffer with 10 to 15 passes or sonicated on ice. The samples were centrifuged at 16,000×*g* for 10 min to remove insoluble materials. To each well, 5 μL of assay buffer was added to measure Fe^2+^ levels and 5 μL of iron reducer was added to measure total iron levels. Then, the cells were incubated for 30 min at 25°C, the iron probe was added, and the cells were again incubated for 60 min at 25°C. Absorbance was measured at 593 nm using a microplate reader (BioTek).

### 2.12 Lipid peroxidation measurement

To evaluate the extent of ferroptosis, lipid peroxidation was assessed to measure the cellular levels of ROS, GSH, MDA, and 4-HNE concentrations in each group. Briefly, SN tissues were homogenized at low temperatures using the lysis solution in the kits. Then, the BCA Assay Kit was used to determine protein concentration. The levels of ROS, GSH, MDA, and 4-HNE were determined following the manufacturer’s protocol.

### 2.13 Statistical analysis

All values were expressed as mean ± SEM of individual samples. Data were analyzed using one-way analysis of variance (ANOVA) followed by Dunnett’s post-hoc test to compare various groups. The level of statistical significance was set at *p* < 0.05. Analyses were carried out using GraphPad Prism 7.03 and SPSS statistical software package, version 20.0.

## 3 Results

### 3.1 EA treatment improved motor dysfunctions in PD model mice induced by MPTP injection

**Figure 1:**
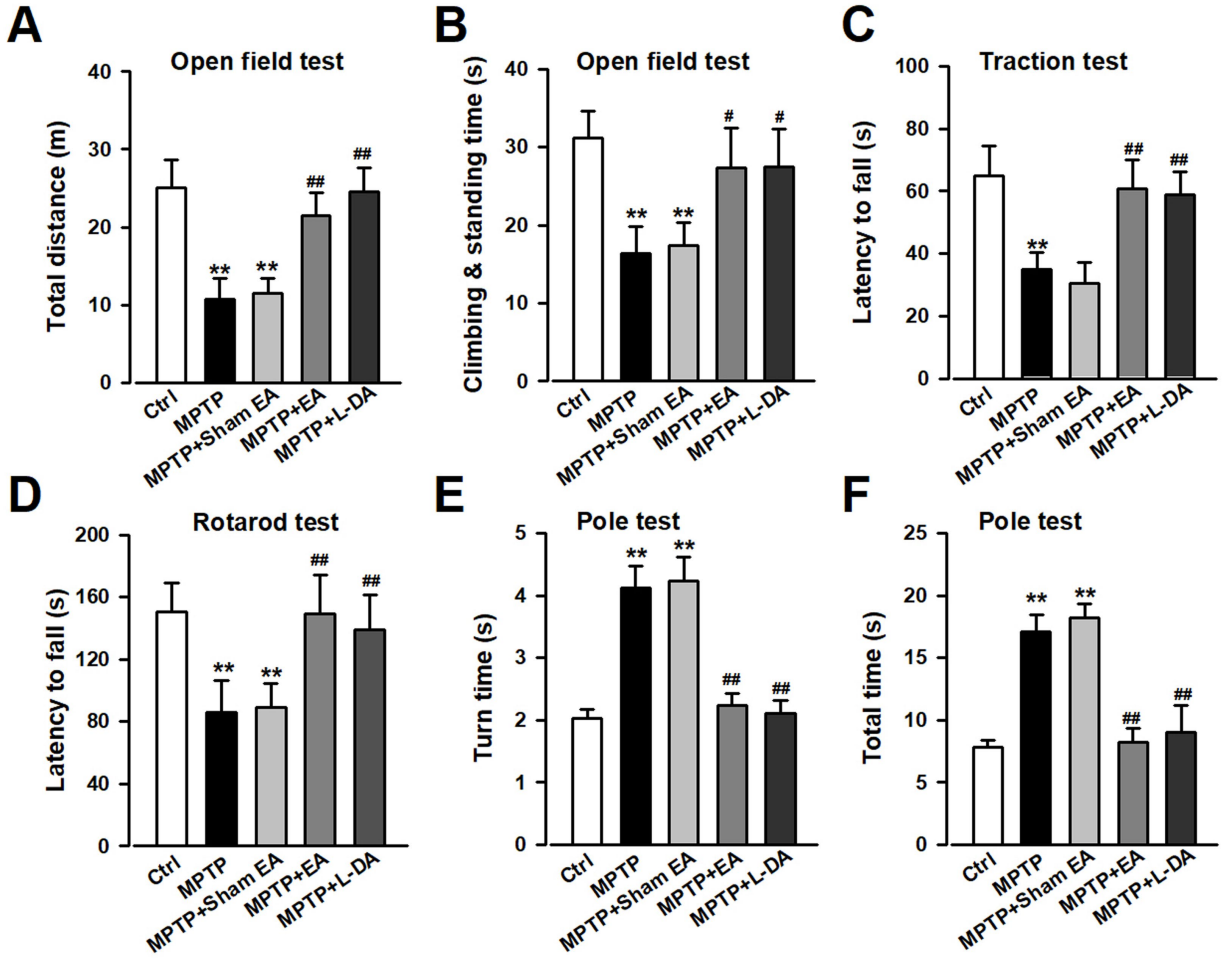
EA treatment improved motor dysfunctions in PD model mice induced by MPTP injection. The motor dysfunctions of mice were determined in Ctrl, MPTP, MPTP + sham EA, MPTP + EA, and MPTP + L-DA groups. **(A,B)** Open field test, **(C)** traction test, **(D)** rotarod test, and **(E,F)** pole test. All data are expressed as mean ± SD (*n* = 8). ^**^p < 0.01 compared with the Ctrl group. ^#^*p* < 0.05; ^##^*p* < 0.01 compared with the MPTP group.

MPTP-induced mice motor dysfunction was used as the PD model in this study, and the therapeutic effects of EA were assessed by conducting behavioral tests, namely OFT, rotarod test, traction test, and pole test. The OFT was carried out first before other behavioral tests. MPTP administration (30 mg/kg, *i.p.*) significantly resulted in defects in the autonomous activity, which was manifested as a decrease in total distance travelled and the climbing and standing time (*p <* 0.01, vs. Ctrl group; Figures 1A,B) during a 5-min period compared with the Ctrl group in the OFT. EA treatment ameliorated the motor dysfunction in PD mice by increasing the total distance (*p <* 0.01, vs. MPTP group; Figure 1A) and the climbing and standing time (*p <* 0.05, vs. Ctrl group; Figure 1B) in the OFT. In accordance with pervasive motor dysfunction, the MPTP insult decreased the latency to fall in the traction test and the average time on the rotarod compared with the Ctrl group (*p <* 0.01, vs. Ctrl group; Figures 1C,D), indicating that MPTP led to an impairment in the motor performance of the front paw and motor coordination, whereas EA treatment improved the motor performance of MPTP mice (*p <* 0.01, vs. MPTP group; Figures 1C,D). In addition, the pole test was used to evaluate the coordination and balancing ability of mice, and the data showed that the MPTP insult increased the turn time and total time (*p <* 0.01, vs. Ctrl group; Figures 1E,F). Furthermore, EA administration decreased the turn time and total time spent in the pole (*p <* 0.01, vs. MPTP group; Figures 1E,F). L-DA administration served as the positive control, and the results from the MPTP + L-DA group (*p <* 0.01, vs. MPTP group; Figure 1) were similar to those of the MPTP + EA group, without a significant difference. These data show that there is no significant improvement in motor function in the sham EA group compared with the MPTP group. Together, these findings indicate that EA at Baihui and Fengfu can effectively reverse the deleterious effects of MPTP on motor function.

### 3.2 EA treatment decreased the loss of DAergic neurons in the SN of PD mice

**Figure 2:**
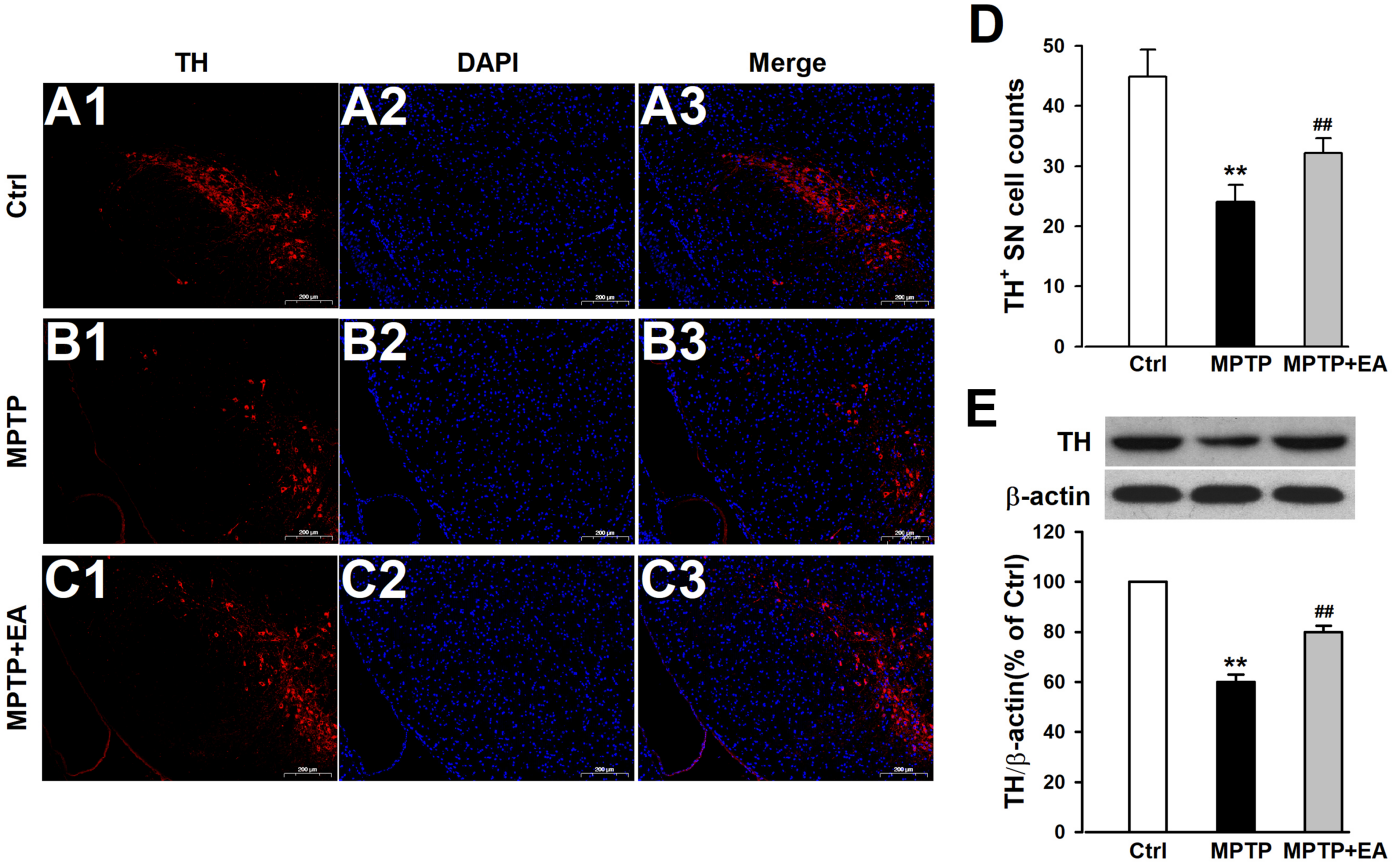
EA treatment decreased the loss of DAergic neurons in the SN of PD mice. **(A–C)** Expression changes of TH in the SN from each group were measured by immunostaining. TH was labeled in red, and the nucleus was labeled in blue. **(D)** TH^+^ SN cells were counted. **(E)** TH protein levels were assessed using Western blot analysis. All data are expressed as mean ± SD (*n* = 3). ^**^p < 0.01 compared with the Ctrl group. ^##^*p* < 0.01 compared with the MPTP group.

The loss of DAergic neurons in the SN is associated with PD symptoms, and TH, the biomarker of DAergic neurons, serves as the rate-limiting enzyme for DA synthesis (Grayson, 2016; Hayes, 2019). Cell counting after immunohistochemical staining for TH-positive (TH^+^) cells in the SN revealed a severe DAergic neuron loss after MPTP exposure compared with the Ctrl group (*p <* 0.01, vs. Ctrl group; Figures 2A–D). EA therapy protected these neurons against MPTP damage (*p <* 0.01, vs. MPTP group; Figures 2A–D). Furthermore, Western blot results showed that the TH protein level in the SN of the MPTP group was decreased compared with that of the Ctrl group (*p <* 0.01, vs. Ctrl group; Figure 2E), and EA treatment significantly attenuated this decrease (*p <* 0.01, vs. MPTP group; Figure 2E). These findings show that EA treatment significantly prevents the loss of DAergic neurons upon MPTP injury. Understanding the mechanism involved in the prevention of DAergic neuron loss can further elucidate the protective mechanism of EA in PD therapy.

### 3.3 EA therapy rescued the decrease in DA neurotransmitters in the SN of PD mice

**Figure 3:**
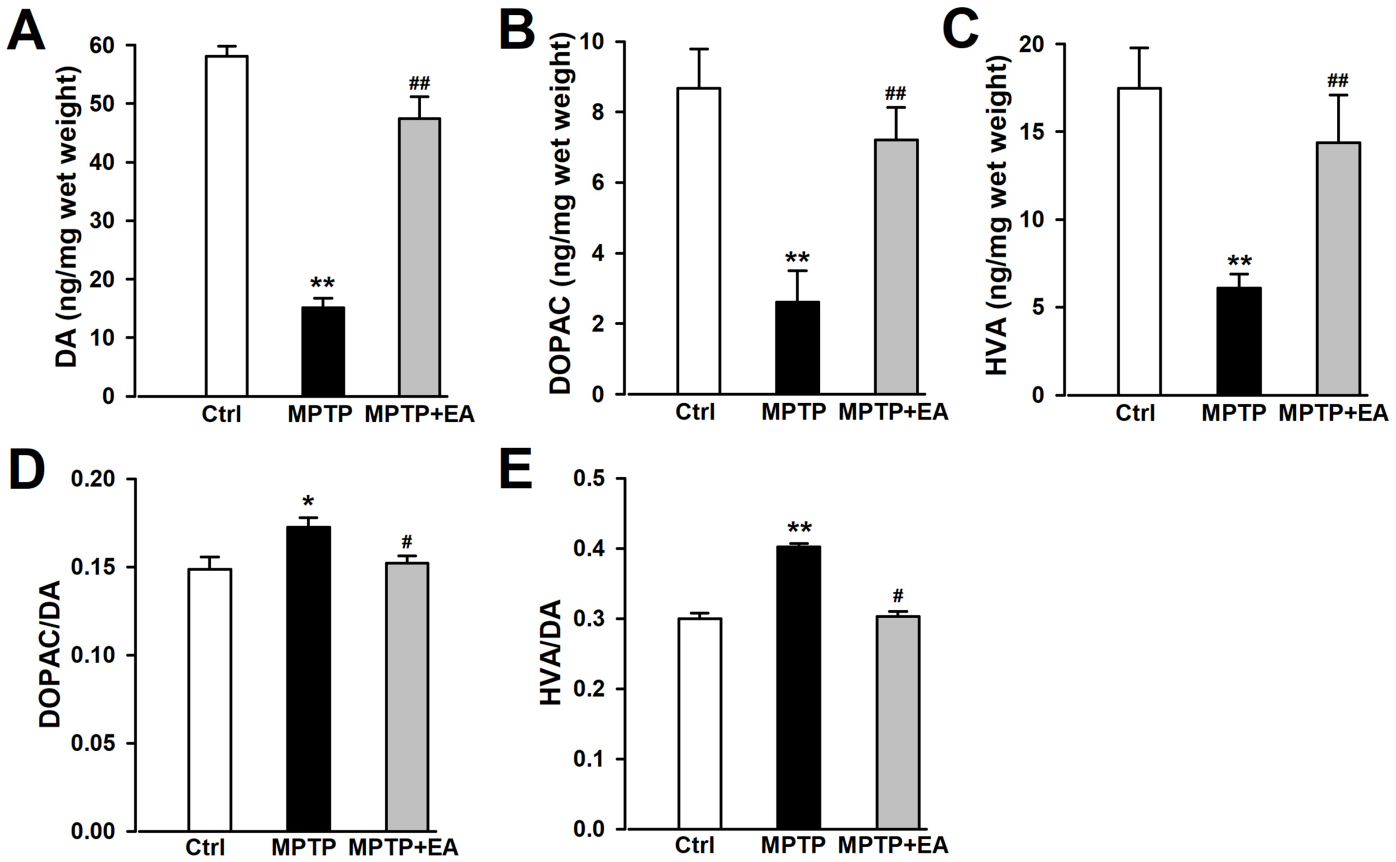
EA therapy rescued the decrease in DA neurotransmitters in the SN of PD mice. **(A–C)** The levels of DA, DOPAC, and HVA were measured using HPLC. TH was labeled in red, and the nucleus was labeled in blue. **(D,E)** DOPAC/DA and HVA/DA ratios were calculated. All data are expressed as mean ± SD (*n* = 6). *p < 0.05; **p < 0.01 compared with the Ctrl group. ^#^*p <* 0.05; ^##^*p <* 0.01 compared with the MPTP group.

The presence of a specific level of neurotransmitters is crucial for proper motor function. The levels of DA and its metabolites DOPAC and HVA in the SN were detected. HPLC analysis revealed that the level of DA in the SN decreased after the MPTP insult (*p <* 0.01, vs. Ctrl group; Figure 3A), which was significantly lower than that of the Ctrl group. In addition, the levels of DOPAC and HVA in the SN also decreased after the MPTP insult (*p <* 0.01, vs. Ctrl group; Figures 3B,C) compared with those of the Ctrl group. These findings suggest that MPTP disturbed DA neurotransmission in the brain, which was characterized by the lower levels of DA and its metabolites DOPAC and HVA. Furthermore, EA administration increased the levels of DA, DOPAC, and HVA in the SN (*p <* 0.01, vs. MPTP group; Figures 3A–C). DOPAC/DA and HVA/DA ratios were calculated based on the estimated DA release and its turnover in the SN. The findings showed that EA treatment can reverse the increased ratios of DOPAC/DA and HVA/DA after MPTP injury (*p <* 0.01, vs. MPTP group; Figures 3D,E), indicating that EA therapy recovered the production of DA after MPTP damage. These findings suggest that EA therapy exerts neuroprotective effects by rescuing DA neurotransmitters, thus improving motor dysfunctions in PD.

### 3.4 Expression levels of ferroptotic proteins GPX4, Nrf2, SLC7A11, and FTH1 were downregulated following MPTP induction in mice and recovered after EA treatment

**Figure 4:**
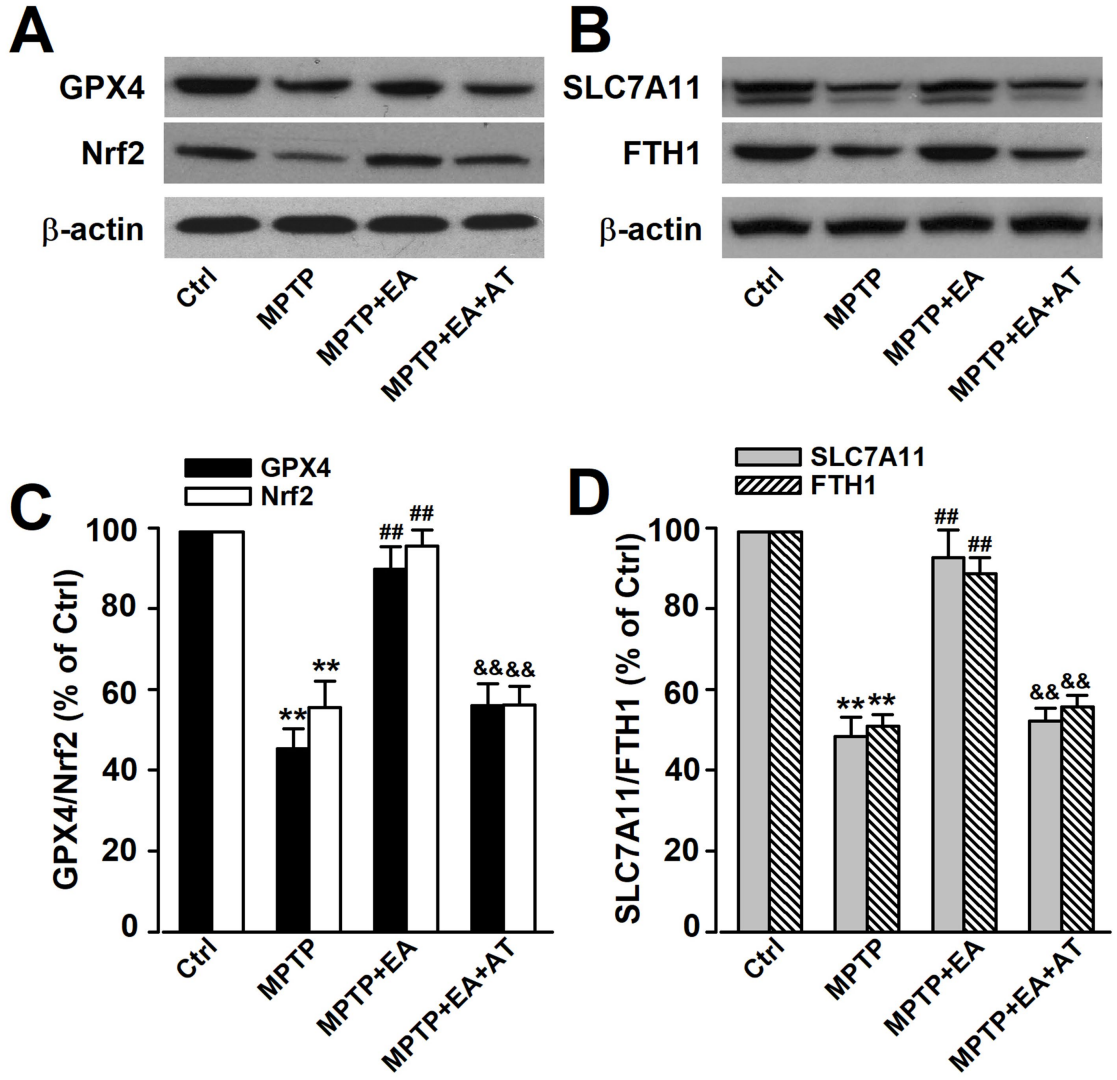
Expression levels of ferroptotic proteins GPX4, Nrf2, SLC7A11, and FTH1 were downregulated following MPTP administration in mice and recovered after EA treatment. **(A,B)** Representative results of Western blot analysis for GPX4, Nrf2, SLC7A11, and FTH1 expression in the SN of mice. **(C,D)** The Nrf2 inhibitor AT reversed the protective effect of EA on the decreased expression of GPX4, Nrf2, SLC7A11, and FTH1 induced by MPTP. All data are expressed as mean ± SD (*n* = 3). ^**^p < 0.01 compared with the Ctrl group. ^##^*p <* 0.01 compared with the MPTP group. ^&&^*p <* 0.01 compared with the MPTP + EA group.

GPX4 and SLC7A11 are key proteins that regulate ferroptosis, which may be an early pathological change in PD. The expression changes of GPX4 and SLC7A11 were measured using Western blot analysis. The results revealed that mice exposed to MPTP showed significantly reduced GPX4 and SLC7A11 expression (*p <* 0.01, vs. Ctrl group; Figures 4A–D), which revealed a positive association between ferroptosis and PD in model mice. The primary iron storage protein FTH1 was involved in iron accumulation in ferroptosis, and a decreased level of FTH1 was observed after the MPTP insult (*p <* 0.01, vs. Ctrl group; Figures 4B,D). These results indicate that ferroptosis occurred in the SN tissue of mice exposed to MPTP. Interestingly, Nrf2 acts as a transcription factor that regulates the expression of ferroptosis-related proteins, including GPX4, SLC7A11, and FTH1, thereby inhibiting the occurrence of ferroptosis (Abdalkader et al., 2018; Chen, 2022). The reduction in the Nrf2 expression level was also observed after MPTP injury (*p <* 0.01, vs. Ctrl group; Figures 4A,C). Furthermore, the decreased expression of GPX4, SLC7A11, FTH1, and Nrf2 was reversed after EA treatment (*p <* 0.01, vs. MPTP group; Figures 4A–D). AT, an inhibitor of Nrf2, was further applied to determine whether EA administration presented anti-ferroptotic effects through regulating the Nrf2/GPX4 axis. The results showed that AT reversed the protective effect of EA on the decreased expression of the aforementioned proteins induced by MPTP, suggesting that EA therapy inhibited ferroptosis by regulating the Nrf2/GPX4 axis, thus alleviating MPTP injury in mice. This led us to preliminarily conclude that EA therapy regulated the Nrf2/GPX4 axis to improve ferroptosis, thus alleviating MPTP injury in mice.

### 3.5 EA treatment restored the levels of lipid peroxidation and iron content in mice SN after MPTP insult

**Figure 5:**
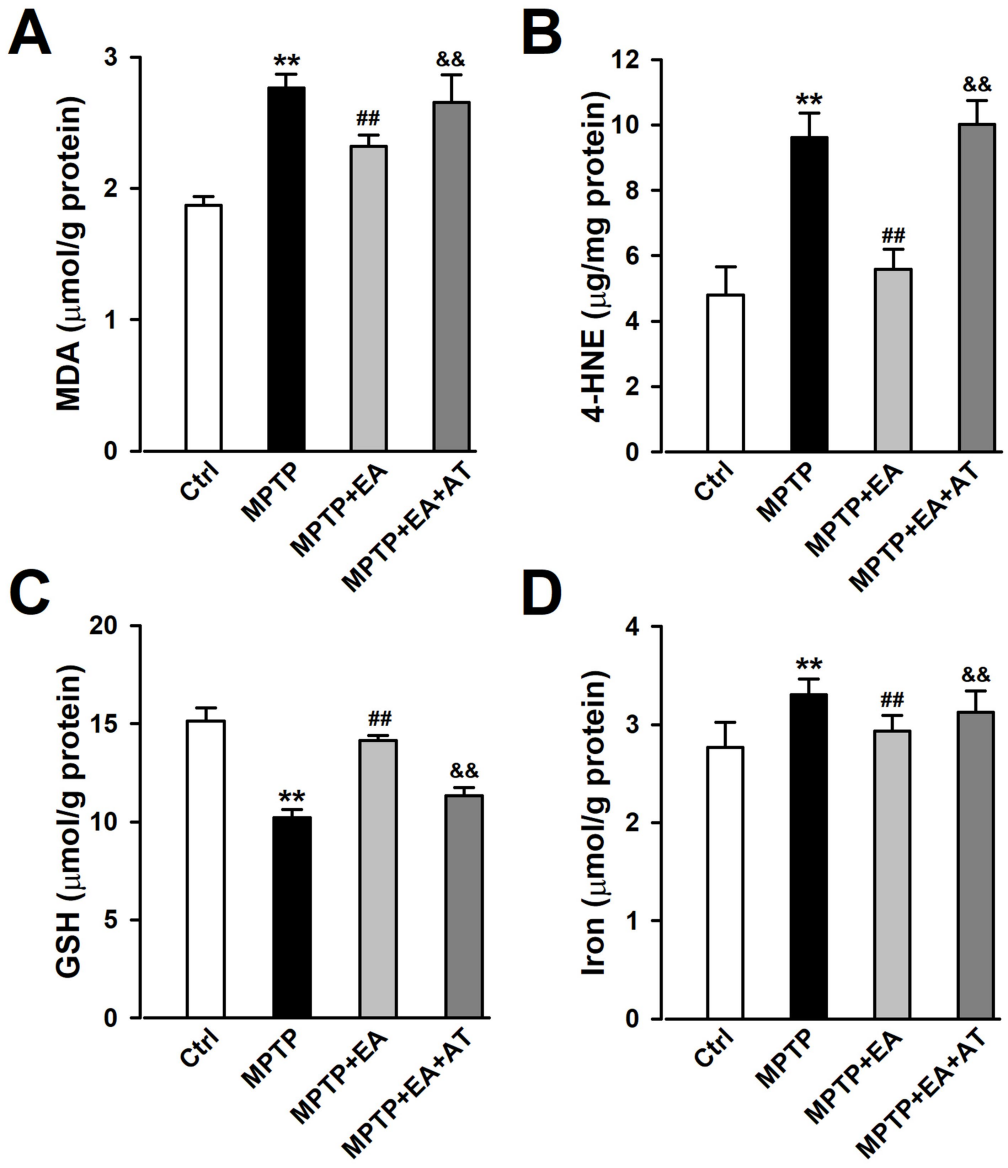
EA treatment restored the levels of lipid peroxidation and iron content in mice SN after MPTP insult. **(A–D)** The expression of MDA, 4-HNE, GSH, and iron in the SN was tested using ELISA kits. ^**^p < 0.01 compared with the Ctrl group. ^##^*p* < 0.01 compared with the MPTP group. ^&&^*p* < 0.01 compared with the MPTP + EA group.

Since ferroptosis was attributable to excessive lipid peroxidation, the level of lipid peroxidation was assessed by detecting the expression of MDA, 4-HNE, and GSH using kits. The results showed that the levels of MDA and 4-HNE increased in the SN after the MPTP insult compared with the Ctrl group (*p <* 0.01, vs. Ctrl group; Figures 5A,B), which indicated a substantial increase in lipid peroxidation levels. However, the expression of GSH was found to be decreased (*p <* 0.01, vs. Ctrl group; Figure 5C). EA treatment decreased the levels of 4-HNE and MDA, but increased the level of GSH in the MPTP group (*p <* 0.01, vs. MPTP group; Figures 5A–C), suggesting that EA treatment can restore the levels of lipid peroxidation and antioxidation upon MPTP injury. Iron is a stimulator of oxidative stress, and iron overload promotes ferroptosis. Thus, the iron content in the SN tissue was also detected using iron assay kits. The results showed that the iron content was significantly increased in the MPTP group compared with the Ctrl group (*p <* 0.01, vs. Ctrl group; Figure 5D), whereas EA treatment decreased the iron content in the MPTP group significantly (*p <* 0.01, vs. MPTP group; Figure 5D), confirming that EA therapy reduces iron accumulation after MPTP injury. However, the inhibition of Nrf2 by AT partially abolished the attenuated lipid peroxidation and iron content after EA therapy. All these findings suggest that EA inhibits ferroptosis by restoring the balance between lipid peroxidation and antioxidation, as well as iron content via Nrf2 activation, thus alleviating MPTP injury in mice. These findings show that Nrf2 modulates PD progression by regulating ferroptosis.

### 3.6 EA therapy corrected mitochondrial dysfunction in mice SN after MPTP insult

**Figure 6:**
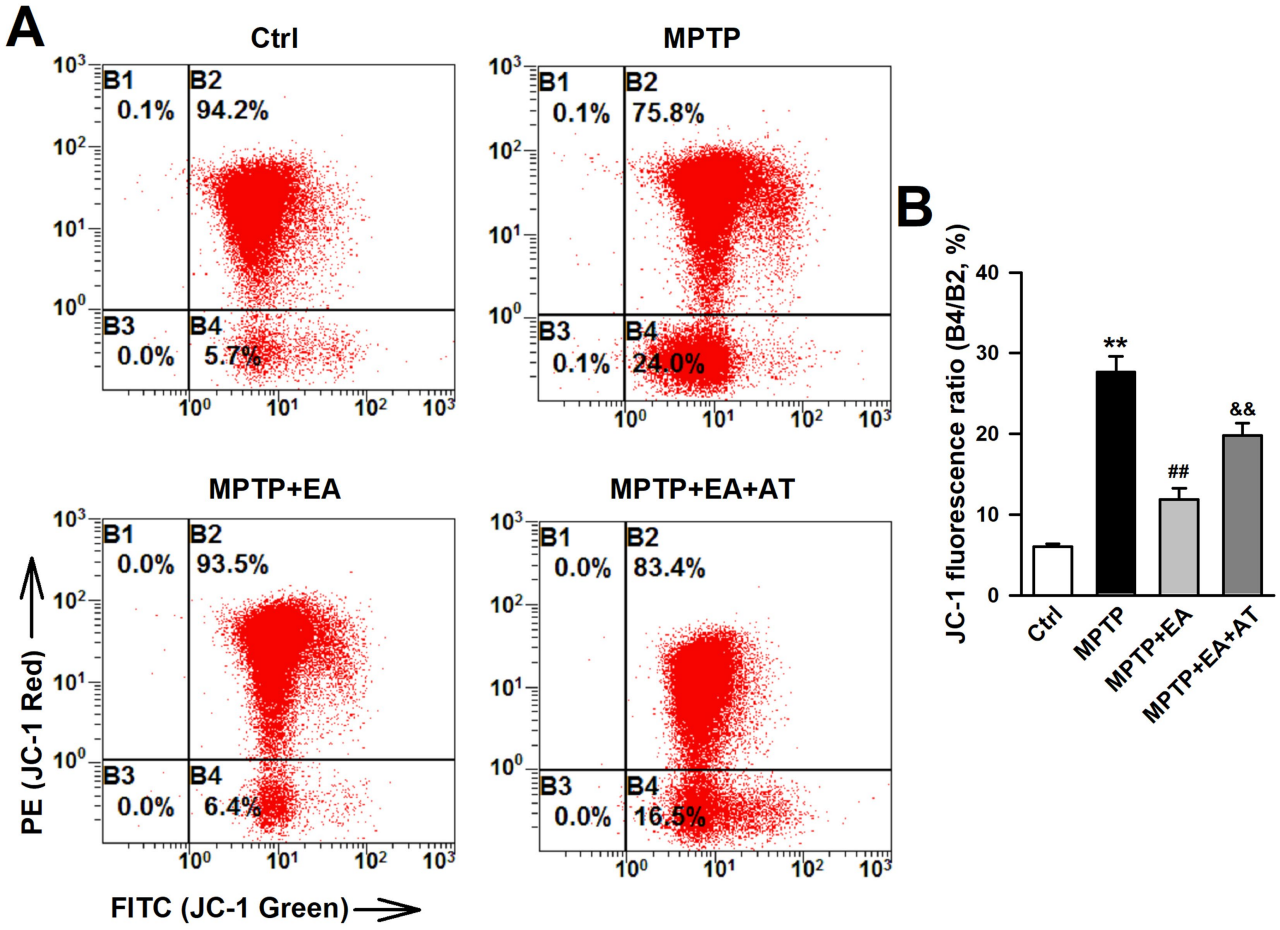
EA therapy corrected mitochondrial dysfunction in mice SN after MPTP insult. **(A)** Scatter diagram of JC-1 staining in neuronal cells using a flow cytometer. **(B)** Quantification of mitochondrial membrane potential in neuronal cells of differently treated mice. (B4/B2 ratio). ^**^p < 0.01 compared with the Ctrl group. ^##^*p* < 0.01 compared with the MPTP group. ^&&^*p* < 0.01 compared with the MPTP + EA group.

Mitochondrial damage also induces ferroptosis in neuronal cells (Abdalkader et al., 2018; Neitemeier et al., 2017). Mitochondrial dysfunction was detected using JC-1 staining after the SN tissue dissociated into single cells. The results indicated that MMP (ΔΨm) was significantly decreased after MPTP damage, as evidenced by an increased monomer/aggregates ratio of JC-1 (*p <* 0.01, vs. Ctrl group; Figure 6). EA therapy improved the decrease in MMP that was induced by the MPTP insult (*p <* 0.01, vs. MPTP group; Figure 6), but the inhibition of Nrf2 by AT partially abolished the MMP improved by EA therapy. Together with the data presented in Figure 6, these findings show that MPTP-induced MDA and 4-HNE accumulation leads to mitochondrial dysfunction. Therefore, activation of Nrf2 protects against mitochondrial damage and rebalances lipid peroxidation and antioxidation upon MPTP injury, which are vital factors associated with ferroptosis induction (Abdalkader et al., 2018; Chen, 2022).

### 3.7 EA therapy alleviated the loss of DAergic neurons in MPTP-induced mice by upregulating Nrf2 expression

**Figure 7:**
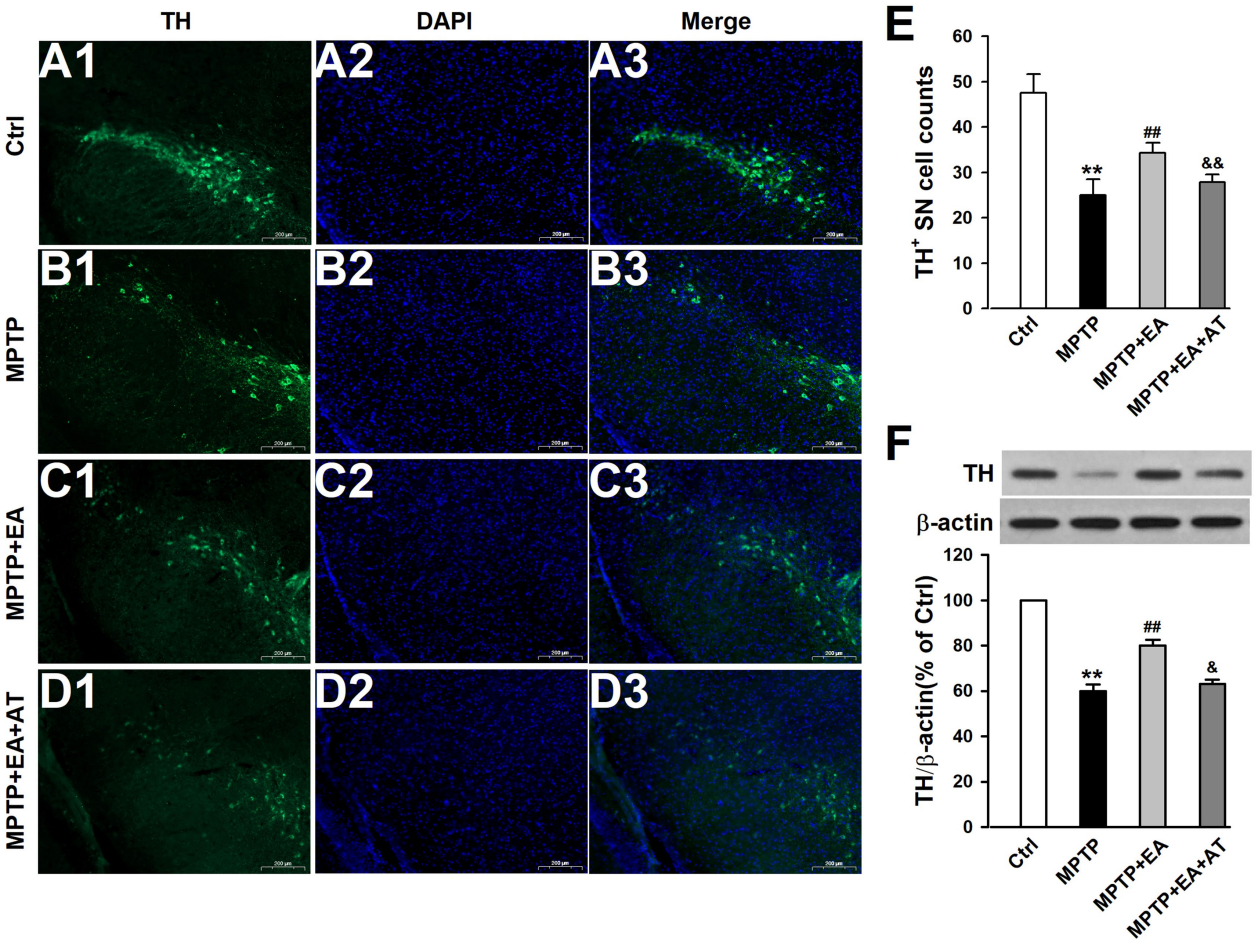
EA therapy alleviated the loss of DAergic neurons in MPTP-induced mice by upregulating Nrf2 expression. **(A–D)** Expression changes of TH in the SN from each group were measured by immunostaining. TH was labeled in green, and the nucleus was labeled in blue. **(E)** TH^+^ SN cells were counted. **(F)** TH protein levels were assessed using Western blot analysis. All data are expressed as mean ± SD (*n* = 3). ^**^p < 0.01 compared with the Ctrl group. ^##^*p* < 0.01 compared with the MPTP group. ^&&^*p* < 0.01 compared with the MPTP + EA group.

The ultimate aim of all beneficial effects of EA therapy is to increase the number of DAergic neurons to restore normal neurotransmission. The findings discussed above indicate that EA therapy improved ferroptosis to ameliorate behavioral deficits in MPTP mice, and hence, we further explored whether EA therapy protected DAergic neurons against MPTP neurotoxicity by regulating the Nrf2/GPX4 signaling pathway. Experiments were carried out in a similar fashion as described earlier. The results showed that EA therapy prevented the loss of TH^+^ cells (*p <* 0.01, vs. MPTP group; Figures 7A–E) and the reduced expression level of TH (*p <* 0.01, vs. MPTP group; Figure 7F), whereas AT, an inhibitor of Nrf2, abolished the protective effects of EA on the reduction in TH^+^ cells (*p <* 0.01, vs. MPTP+EA group; Figures 7A–E) and the decreased expression of TH protein (*p <* 0.05, vs. MPTP+EA group; Figure 7F) induced by MPTP. This finding confirms that EA treatment inhibited ferroptosis by regulating the Nrf2/GPX4 axis, thus protecting DAergic neurons in the SN against MPTP injury in mice.

## 4 Discussion

The findings of this study showed that EA treatment significantly ameliorated motor dysfunctions in mice induced by MPTP insult. Mechanically, EA administration ameliorated MPTP-evoked ferroptosis and mitochondrial injury by promoting the survival of DAergic neurons and increasing the neurotransmission of DA to provide symptomatic relief in the MPTP-induced PD model. The activation of the Nrf2/System Xc-/GPX4 pathway was responsible for EA-mediated anti-ferroptosis effects that promoted the survival of DAergic neurons in the SN subjected to the MPTP insult. Furthermore, AT, an inhibitor of Nrf2 activation, abolished the inhibition of ferroptosis and cell mitochondrial injury mediated by EA therapy in PD mice upon MPTP stimulation. These results highlight that MPTP-evoked ferroptosis and mitochondrial injury are responsible for the pathogenesis of the PD model and that EA can be used as a potential therapeutic strategy for PD treatment as it suppresses ferroptosis.

The primary pathophysiological feature of PD is the degeneration of DAergic neurons in the SN (Dionisio et al., 2021; Lu et al., 2023), and a specific DAergic neuroprotective drug for PD therapy has not been developed. A better understanding of the pathological mechanisms associated with DAergic neuronal loss is required for designing novel therapeutic targets for PD treatment. EA, a modification to conventional acupuncture, offers therapeutic effects to central nervous system diseases, including PD, by promoting neuronal survival; however, the mechanisms involved in EA are still not well understood. Consistent with previous findings, our experimental results revealed that EA significantly alleviates MPTP-induced motor dysfunctions in mice (Figure 1) by decreasing the loss of DAergic neurons (Figure 2). Our findings also showed that ferroptosis is an iron-dependent regulatory cell death driven by lipid peroxidation, which is involved in the loss of DAergic neurons in the pathogenesis of PD. Therefore, developing an effective method for PD treatment against ferroptosis is critical. Our findings showed the therapeutic effects of EA are attributable to anti-ferroptosis, which are manifested as the increased levels of ferroptosis-related proteins Nrf2, SLC7A11, GPX4, and FTH1 (Figure 4); decreased levels of intracellular MDA, 4-HNE, and iron; and an increased level of GSH (Figure 5). These findings show the association between EA therapy and anti-ferroptosis against PD. Elucidating how ferroptosis provokes PD will reveal new therapeutic opportunities to treat PD.

Iron plays a crucial role in myelination, neurotransmitter biosynthesis, and energy metabolism (Belaidi and Bush, 2016; Crichton et al., 2011); therefore, cellular iron homeostasis plays an important role in neuronal function, which is highly regulated by iron uptake, distribution, and efflux (Ward et al., 2014; Weinreb et al., 2013). A previous study has shown that intracellular iron accumulation has toxic effects in terms of biological function and generates a large amount of oxygen free radicals, which in turn damage intracellular DNA, proteins, and cell organelles, especially mitochondria (Stockwell et al., 2017). Our study also found that the MMP of SN dopaminergic neurons decreased after the administration of MPTP (Figure 6). Iron dysregulation can block mitochondrial electron transport chain activity and generate ROS, which increases oxidative stress. Our results showed an increased iron level and decreased expression of FTH1, the primary iron storage protein, which was involved in iron accumulation in ferroptosis after the MPTP insult (Figure 4), suggesting that abnormal iron homeostasis induces ferroptosis. Furthermore, iron deficiency or accumulation can lead to cellular dysfunction and energy crisis.

The increased Fe^2+^ level is a notable maker for ferroptosis, and intracellular iron accumulation triggers the depletion of GSH, leading to a decrease in GPX4 activity and the inability to metabolize lipid peroxides, thus resulting in free Fe^2+^-oxidizing lipids through the Fenton reaction to produce ROS and leading to ferroptosis (Dixon et al., 2012). Free Fe^2+^ accelerates the aggregation of *α*-synuclein, leading to the death of DAergic neurons (Do Van et al., 2016). Numerous studies have shown that there is an increase in free iron storage and lipid peroxidation levels in the SN of PD patients. Recent studies have further confirmed that the inhibition of ferroptosis in DAergic neurons attenuates motor dysfunctions in MPTP-induced mice (Guiney et al., 2017). Consistent with previous findings, our results revealed that EA stimulation at Baihui and Fengfu significantly inhibited ferroptosis and attenuated MPTP-induced motor dysfunctions in mice. In addition, in MPTP-treated DAergic neurons, MPTP caused mitochondrial damage, which was manifested as a decrease in MMP. EA treatment can improve the mitochondrial function of DAergic neurons. Nrf2 is a key regulatory factor for maintaining oxidative stability in cells and is associated with ferroptosis. Genes related to iron overload and lipid peroxidation are the target genes of Nrf2. Previous studies have reported that specific knockout of the Nrf2 gene reduces the expression of SLC7A11, inhibits GPX4 activity, and enhances the sensitivity of erastin-induced ferroptosis in PC12 cells. Regarding whether Nrf2 is involved in regulating the transmission of stimulatory signals at SNc DAergic neurons, our findings further confirmed that the administration of the Nrf2 antagonist AT partially attenuated the EA-triggered increase in MMP. These results further demonstrated that EA stimulation exerts a neuroprotective effect through Nrf2-mediated inhibition of MPTP-induced ferroptosis.

In conclusion, our results revealed for the first time that EA stimulation at Baihui and Fengfu of MPTP-treated mice inhibited the ferroptosis of DAergic neurons through activation of Nrf2, attenuated oxidative stress and mitochondrial dysfunction, and alleviated MPTP-induced motor dysfunctions. However, the safety and side effects of EA in PD patients should be further emphasized. Although clinical studies have demonstrated the safety and minimal side effects of EA in PD patients, it should be noted that individual differences, such as disease stage and acupoint sensitivity, may affect treatment outcomes. To address this challenge, future clinical studies should standardize EA parameters and stratify patient populations.

## Data Availability

The datasets presented in this study can be found in online repositories. The names of the repository/repositories and accession number(s) can be found in the article/supplementary material.
